# Isolation of Two Isochlorogenic Acid Isomers from Phenolic Rich Fraction of *Artemisia* *turanica* Krasch. 

**DOI:** 10.22037/ijpr.2019.15182.12919

**Published:** 2020

**Authors:** Fahimeh Moradi-Afrapoli, Ghazal Saremi, Sajjad Nasseri, Seyed Ahmad Emami, Mahdi Mojarrab

**Affiliations:** a *Division of Pharmaceutical Biology, Department of Pharmaceutical Sciences, University of Basel, Basel, Switzerland. *; b *Students Research Committee, Kermanshah University of Medical Science, Kermanshah, Iran.*; c *Pharmaceutical Sciences Research Center, Health Institute, Kermanshah University of Medical Sciences, Kermanshah, Iran. *; d *Department of Pharmacognosy, School of Pharmacy, Mashhad University of Medical Sciences, Mashhad, Iran.*

**Keywords:** Artemisia turanica, Asteraceae, Dicaffeoylquinic acid, Isochlorogenic acid, Antioxidant activity, Total phenolic content

## Abstract

Total phenolic content (TPC) and antioxidant capacity of five different extracts (petroleum ether (40-60), dichloromethane, ethyl acetate, ethanol and ethanol-water (1:1 v/v)) of* Artemisia turanica (A. turanica)* aerial parts were determined and phytochemical study on the most promising extract was carried out. Folin–Ciocalteu method, 1, 1-diphenyl-2-picrylhydrazyl (DPPH) radical scavenging test, β-carotene bleaching (BCB) method, and ferrous ion chelating (FIC) assay were performed. Vacuum liquid chromatography (VLC) and semi-preparative HPLC were used for bioassay-guided phytochemical isolation. Structures of isolated compounds were established using spectroscopic analysis including NMR and MS. Among all the extracts analyzed, the hydroethanolic extract exhibited the highest phenolic content and antioxidant activity. VLC of this extract yielded seven fractions (A to G) which were subjected to all antecedent experiments. The same sample (Fraction D) showed the highest total phenolic content and free radical scavenging activity but the only statistically significant correlation between TPC and EC_50_ values was observed for BCB. 3,5-dicaffeoylquinic acid (isochlorogenic acid A), and 4,5-dicaffeoylquinic acid (isochlorogenic acid C) was isolated from the most active fraction. Antioxidant activity of *A. turanica* is probably partly due to the presence of isomers of isochlorogenic acid.

## Introduction

The genus *Artemisia*, belonging to tribe Anthemideae (family Asteraceae), contains the well-known medicinal plants. Different species could be found in the temperate zone of Asia, Europe, and North America (1). About 34 *Artemisia* species have been identified in Iran (2). *A. turanica* Krasch. (locally named “Dermaneye Ghermez”) which grows naturally in the northeastern region of the country is one of these species (3). 1,8- cineole, chrysanthenone, and davanone have been reported as major constituents of the essential oil of the aerial parts while another study has shown a notable increase of α-thujone content in the leaf essential oil (4, 5). Methanolic extract of the aerial parts of the plant has been effective against *BacillusSubtilis, Staphylococcus aureus* and *Pseudomonas aeruginosa* (6). Moderate toxicity of crude hydroethanolic extract against HepG2 cell line as well as the moderate effect of the ethanolic extract on *in-vitro *leishmanicidal activity have been reported (7, 8). There are some reports on the antimalarial activity of *A. turanica *extracts as well (9-11). While the essential oil and different extracts of *A. turanica* have shown antioxidant and cytotoxic activity, a recent study has demonstrated the *in-vitro *neuroprotective potential of *A. turanica *(12-14)*. *As per the previous reports enclosing the diverse beneficial effects of *A. turanica*, the authors have been stimulated to study the antioxidant effect of this species using different assays. To the best of our knowledge, although collectively these studies suggested the therapeutic benefits of this medicinal plant, there is no comprehensive study on the free radical scavenging and ferrous ion chelating activities of different extracts and fractions. This study aims to investigate the anti-oxidative role of different extracts/ fractions of *A. turanica* using different assays and examine whether their activity is correlated with the phenolic content. Another aim of the present work is to determine the structure of phytochemicals which are responsible for the observed effects.

## Experimental

*Chemicals*

Gallic acid, Linoleic acid, sodium carbonate, ferrous chloride, dimethyl sulfoxide (DMSO), chloroform, ethylenediaminetetraacetic acid (EDTA), Tween^®^ 40, Folin-Ciocalteu’s phenol reagent, butylated hydroxytoluene (BHT), LiChroprep^®^ RP-18 (15-25 µm) were purchased from Merck. 1,1-diphenyl-2-picrylhydrazyl (DPPH) and β- Carotene were purchased from Sigma- Aldrich, ascorbic acid from VWR, ferrozine iron reagent from Acros Organics and all the solvents used for extraction and purification procedures were of gradient grade and purchased from Scharlau (Spain) and Caledon (Canada).

*General experimental procedures*

The chromatographic system for semi-preparative HPLC consisted of a binary pump YL 9111S, a PDA detector YL9160 and a VertiSep UPS C18 (250 × 30 mm i. d., 10 μm) column. An Ascentis^®^ (250 ×10 mm i. d., 5 μm) column was replaced for final purification. NMR spectra were recorded on a Bruker AVANCE III 500 MHz spectrometer in dimethyl sulfoxide-d6 as the solvent and the residual solvent signal was used as internal standard. ESIMS data were obtained on an Esquire 3000 plus ion trap mass spectrometer (Bruker).

*Plant material*

Aerial parts of *A. turanica *Krasch were collected from Samie Abad, Torbat- e Jam (Razavi Khorasan province, northeast of Iran) in September 2010. The identity of the plant sample was confirmed by Dr. V. Mozaffarian (Research Institute of Forest and Rangelands, Tehran, Iran). Voucher number 12572 is retained in the herbarium, Department of Pharmacognosy, Faculty of Pharmacy, Mashhad University of Medical Sciences, Mashhad, Iran.

*Preparation of extracts*

Air-dried and ground aerial parts (200 g) of* A. turanica *were extracted with petroleum ether (40-60), dichloromethane, ethyl acetate, ethanol and ethanol-water (1:1 v/v), respectively (Sequential maceration with ca. 3 × 2 L of each solvent). The extracts were filtrated with filter paper and dried using rotary evaporator at reduced pressure at a temperature below 45 °C to yield 5.51, 24.23, 1.21, 7.78, and 37.50 g of each extract, respectively.

*Chromatographic Fractionation and Isolation*

Reversed-phase high performance liquid chromatography (RP-HPLC) is now commonly used for the separation of complex the mixtures of phenolic compounds and the other natural products (15). Fifteen grams of the hydroethanolic extract which exhibited better results in two different antioxidant assays was subjected to reversed-phase VLC using a step gradient of MeOH-H_2_O (0.5:9.5, 1:9, 2:8, 4:6, 6:4, 8:2, 10:0) to give seven fractions (A, B, C, D, E, F and G) respectively (Table 1). A portion (915 mg) of fraction D (eluted by 40% methanol in water) was re-fractionated by semi-preparative HPLC (mobile phase: 0–30 min, MeOH from 25 to 55% in H_2_O; 30–31 min MeOH from 55 to 100% in H_2_O; 31–35 min 100% MeOH, flow rate 8 mL/min) to yield six subfractions. Further purification of the subfraction4 (93.9 mg, *t*R = 20.4 min) by semi-preparative HPLC (mobile phase: 0–20 min, MeOH from 30 to 50% in H2O; 20–25 min 50% MeOH, 25–26 min MeOH from 50 to 100% in H2O; 26–30 min 100% MeOH, flow rate 3 mL/min) yielded compounds **1 **(12.2 mg, *t*R = 16.6 min) and **2 **(5.4 mg, *t*R = 20.4 min). The structures of isolated compounds were elucidated using spectroscopic analysis including ESIMS, ^1^H- and 2D-NMR.

*Total phenolic contents*

The total phenolic content (TPC) was measured by the Folin–Ciocalteu method with some modifications (16, 17). Different concentrations of samples in water (0.500 mL) were mixed with 2.5 mL of Folin- Ciocalteu reagent (0.2 N). Two mLof Na_2_CO_3 _solution (75 g/L) was added after 5 min. After 2 h standing in the dark, the optical density was measured at 760 nm against a blank. The total phenolic contents were calculated based on the calibration curve of gallic acid and expressed as milligrams of gallic acid equivalents (GAE), per gram of the dried samples.

*DPPH radical scavenging activity*

The assay was performed according to the method of Hatano *et al*. with slight modifications (18). Briefly, test samples were dissolved in methanol at different concentrations. Equal volumes of 0.2 mM solution of DPPH in methanol were added to each of the test tubes. The mixture was shaken vigorously and maintained in the dark for 30 min. Then, the absorbance was read at 517 nm against a blank. Butylated hydroxytoluene (BHT) and ascorbic acid were used as standard references. The scavenging activity was calculated using the formula:

I% = (Ac – A)/Ac × 100

Where A_c_ = absorbance of the control and A = absorbance of a tested sample in 30 min.

*Metal chelating activity*

The chelating activity of the extracts and fractions for ferrous ions Fe^2+ ^was measured adopting the ferrous iron– ferrozine complex method with some modification (19). Briefly, 25 µL of FeCl_2 _solution (2 mM) was added to a mixture containing 2 mL of methanolic solution of test sample and 1.5 mL of H_2_O. The reaction was started by adding 50 µL of ferrozine solution (5 mM) to each test tube after 30 seconds. The mixtures were shaken well and incubated for 10 min at room temperature. The absorbance of the solution was then read at 562 nm. EDTA and quercetin were used as positive controls. The ability of the samples to chelate ferrous ion was calculated using the equation mentioned above for DPPH radical scavenging activity.

*Inhibition of* *β* *-* *carotene bleaching*

Antioxidant potential of the extracts and fractions was determined by a slightly modified version of the β-carotene bleaching method (20). Linoleic acid (33 µL) was added to 225 mg of Tween 40 and 750 µL of β-carotene solution (0.500 mg/mL). The solvent was completely removed using a rotary evaporator. After adding 75 mL of oxygenated distilled water, the mixture was emulsified for 15 min in a sonicator to give emulsion A. Aliquots of 3.5 mL of this emulsion were transferred into a series of stopper test tubes containing 1 mL of the samples dissolved in water or DMSO in different concentrations. Optical density (OD) at 470 nm was recorded for all the samples immediately (t = 0) and at the end of the assay time (t = 120). An emulsion which consisted of 50 mL of oxygenated water, 22 µL of linoleic acid and 150 mg of Tween 40 was also prepared to be used as the blank to zero the spectrophotometer. The percentage of inhibition was calculated according to the following formula: 

I% = (A_A (120)_ – A_C (120)_)/(A_C (0)_ – A_C (120)_) × 100

Where A_A (120) _is the absorbance of the sample at t = 120 min, A_C (120)_ is the absorbance of the control at t = 120 min, and A_C (0) _is the absorbance of the control at t = 0 min.

*Statistical analysis*

All the experiments were performed in triplicate. The data were reported as mean ± standard deviation (SD) (n = 3) and evaluated by non- parametric Friedman test. The difference was considered to be statistically significant if *P* < 0.05. Pearson’s correlation coefficients (r) between the total phenolic contents of the samples and calculated EC_50_ values were determined in each antioxidant assay.

## Results

*Extraction and isolation*

**Figure 1:**
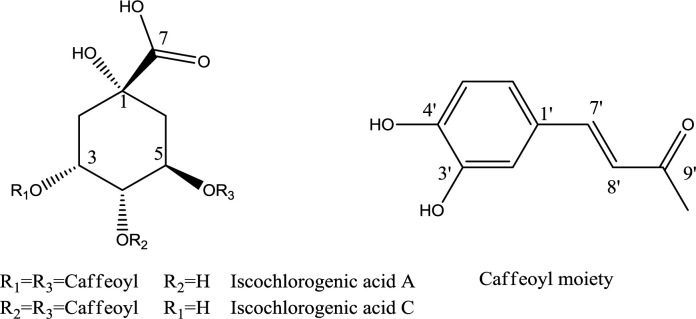
Chemical structures of isolated compounds

Fractionation of the hydroethanolic extract by a combination of VLC and semi-preparative HPLC on RP-18 afforded compounds **1 **and **2 **(Figure 1). The chemical structures of isolated compounds were elucidated unequivocally through ESIMS and NMR, and also all spectroscopic data were in agreement with previously published data (21-24).

Compound **1** (3,5- dicaffeoylquinic acid): brown powder. ESI-MS (m/z): 515.2 [M-H]^-^, 1031.5 [2M-H]^-^. ^1^H NMR (500 MHz, DMSO-d_6_) δ (ppm): 1.99- 2.15 (4H, m, H-2 and H-6), 3.84 (1H, m, H-4), 5.18 (1H, m, H-5), 5.22 (1H, m, H-3),6.18 (1H, d, *J *= 16.0 Hz, H-8”);6.25 (1H, d, *J *= 16.0 Hz, H-8’); 6.78 (1H, overlapping signals (ov), H-5”), 6.79 (1H, ov, H-5’), 6.98 (1H, ov, H-6”), 6.99 (1H, ov, H-6’),7.05 (1H, br s, H-2”), 7.06 (1H, br s, H-2’), 7.45 (1H, d, *J *= 16 Hz, H-7”), 7.49 (1H, d, *J *= 16 Hz, H-7’); ^13^C-NMR (data from HSQC and HMBC spectra, DMSO-d6) δ (ppm): 35.2 (C-2), 36.4 (C-6), 68.4 (C-4), 71.3 (C-3 and C-5), 72.4 (C-1), 114.9 (C-8”), 115.0 (C-2’), 115.1 (C-2”), 115.3 (C-8’), 116.2 (C- 5’ and C-5″), 121.6 (C-6′ and C-6”), 125.3 (C-1’), 125.5 (C-1”), 145.2 (C-7′ and C-7″), 145.6 (C-3′ and C-3″), 148.4 (C-4’), 148.6 (C-4”), 165.6 (C-9”), 166.2 (C-9’), unobserved signal (C-7).

Compound **2** (4, 5-dicaffeoylquinic acid): brown powder. ESI-MS (m/z): 515.2 [M-H]^-^, 1031.5 [2M-H]^-^. ^1^H NMR (500 MHz, DMSO-d_6_) δ (ppm): 2.00- 2.20 (4H, m, H-2 and H-6), 4.20 (1H, m, H-3), 4.97 (1H, m, H-4), 5.43 (1H, m, H-5), 6.14 (1H, d, *J *= 16.0 Hz, H-8”);6.23 (1H, d, *J *= 16.0 Hz, H-8’); 6.75 (2H, d, *J *= 7.6 Hz, H-5’ and H-5”), 6.95 (1H, ov, H-6”), 6.97 (1H, ov, H-6’), 7.02 (2H, br s, H-2’ and H-2”), 7.43 (1H, d, *J *= 16.0 Hz, H-7”), 7.48 (1H, d, *J *= 16.0 Hz, H-7’); ^13^C-NMR (data from HSQC and HMBC spectra, DMSO-d6) δ (ppm): 37.5 (C-2), 38.0 (C-6), 67.1 (C-3), 68.1 (C-5), 74.0 (C-4), 74.2 (C-1), 114.1 (C-8”), 114.4 (C-8’), 115.2 (C-2’ and C-2”), 115.9 (C- 5’ and C-5″), 121.7 (C- 6’ and C-6″), 125.6 (C-1’ and C-1”), 145.7(C-7′ and C-7″), 145.7 (C-3′ and C-3″), 148.5 (C-4’ and C-4”), 166.0 (C-9”), 166.4 (C-9’), unobserved signals (C-1 and C-7).

*Total Phenolic Content*

Regression equation of the calibration curve of gallic acid (R^2^ = 0.997, y = 0.011x + 0.057) was used to calculate the content of phenolics and expressed in GAE as milligrams per gram of each sample (mg GAE/g extract or fraction). Large variations in TPC of the samples were found, ranging from 24.36 ± 1.55 (fraction A) to 255.00 ± 10.29 (fraction D) mg GAE/g fraction (Table 1).

*DPPH radical scavenging activity*

All the samples except fraction A and three extracts (petroleum ether, dichloromethane, and ethyl acetate) showed moderate to strong scavenging activity on the DPPH radical. The highest activity was recorded for ethanolic extract, with the EC_50_ value of 18.31 ± 0.59 µg/mL, followed by the fraction F and hydroethanolic extract with the EC_50_ values of 18.43 ± 0.45 and 20.13 ± 1.07 µg/mL, respectively (Table 1).

*Metal chelating activity*

The highest ferrous ion chelating effect among the samples was shown by fraction B, with the EC_50_ value of 28.96 ± 3.23 µg/mL followed by hydroethanolic extract and fraction A with the EC_50_ values of 47.88 ± 4.72 µg/mL and 47.92 ± 19.35 µg/mL, respectively (Table 1). While fractions C to G had moderate activity, the other extracts did not show any remarkable color changes, although decreases in absorbance readings- except petroleum ether extract- were recorded.

*Inhibition of *β-*carotene bleaching*

Fraction D showed the best inhibitory performance in BCB assay, with an EC_50_ value of 4.92 ± 1.11 μg/mL while Fraction B (EC_50_= 98.17 ± 0.17 μg/mL) exhibited the lowest (Table 1).

*Statistical analysis*

Pearson’s correlation coefficients between TPC and calculated EC_50_ values for DPPH, FIC and BCB assays took the values of -0.531, -0.032, and -0.696, respectively. The lowest correlation was seen between the TPC of the samples and their ability to chelate ferrous ions. No significant correlation was observed between TPC and DPPH radical scavenging activities of the samples as well. The highest correlation between the results of BCB assay and total phenolic contents was observed. The results of Friedman test showed no significant difference in the assays in screening the samples for their antioxidant ability.

**Table 1:** Antioxidant performance and total phenolic contents (TPC) of petroleum ether (PE), dichloromethane (DCM), ethyl acetate (EA), ethanol (EtOH) and ethanol/water (EtOH/Wt) extracts and different fractions (A-G) of the ethanol/water extract of *A. turanica*

**Sample**	**Extraction/ fractionation yield (g)**	**EC**_50_ **(µg/ mL)**			**TPC** **(mg GAE /g)**
		**DPPH assay**	**FIC assay**	**BCB assay**	
PE	5.51	355.74 ±132.85	n.s.	53.24 ± 2.93	43.64 ± 14.20
DCM	24.23	463.49 ± 117.26	585.11 ± 16.12	28.42 ±0.27	75.76 ± 11.22
EA	1.21	101.53 ± 15.95	916.34 ± 49.84	22.13 ± 6.03	106.67 ± 1.39
EtOH	7.78	18.31 ± 0.59	905.19 ± 340.13	11.29 ± 1.29	154.42 ± 3.03
EtOH/Wt	37.50	20.13 ± 1.07	47.88 ± 4.72	8.14 ± 1.92	136.00 ± 3.47
Fr. A	1.86	113.79 ± 16.17	47.92 ± 19.35	81.43 ± 13.67	24.36 ± 1.55
Fr. B	1.19	50.90 ± 4.85	28.96 ± 3.23	98.17 ± 0.17	77.58 ± 0.95
Fr.C	0.82	46.77 ± 4.94	65.86 ± 15.59	65.90 ± 1.83	98.94 ± 0.26
Fr. D	3.01	21.99 ± 1.82	118.36 ± 9.49	4.92 ± 1.11	255.00 ± 10.29
Fr. E	3.79	20.30 ± 3.34	53.33 ± 4.83	23.83 ± 0.07	169.85 ± 4.12
Fr. F	2.42	18.43 ± 0.45	80.91 ± 4.32	15.19± 3.41	148.48 ± 4.10
Fr. G	1.04	37.41 ± 1.74	194.76± 26.41	16.88 ± 2.54	104.24 ± 1.60
BHT	---	4.96 ± 0.66	---	0.469 ± 0.22	---
Vit C	---	4.74±0.19	---	---	---
EDTA	---	---	18.94 ± 2.88	---	---
Quercetin	---	---	88.35 ± 4.09	---	---

DPPH: 1, 1-diphenyl-2-picrylhydrazyl.

FIC: ferrous ion chelating.

BCB: β-carotene bleaching.

GAE: gallic acid equivalents.

## Discussion

To the best of our knowledge, this is the first report on the presence of two dicaffeoylquinic acid isomers (isochlorogenic acids A and C) in *A. turanica.* The structures of the isolated compounds were elucidated by ESIMS, ^1^H-, and 2D-NMR. Both compounds showed the same pseudo-molecular-ion peak at m/z 515.2 ([M-H]^-^), in their ESI-MS, representing the molecular formula C_25_H_24_O_12_. The ^1^H NMR spectrum of the compounds revealed the presence of two caffeoyl moieties with *trans*-geometries of the double bonds (J = 16.0 Hz). The HMBC correlations between H-atoms of quinic acid (H-3, H-4, and H-5) and C-9’ or C-9” were used to determine the linkage positions of caffeoyl groups. The signals of the H-atoms (H-3, H-4, and H-5) in compound **1** were observed between 3.84 and 5.22 ppm, while the same H-atoms in compound **2** resonated between 4.20 and 5.43 ppm. The upfield protons in these areas were H-4 and H-3, in compounds **1** and **2**, respectively. In comparison with the corresponding carbon atom in compound **1**, a downfield shift was observed for C-2 in compound **2**. These facts, along with the comparison of the rest of spectroscopic data with those reported in the literature, allowed identification of the compounds **1** and **2** as 3,5-di-O-caffeoylquinic acid (3,5-di-CQA), and 4,5-di-O-caffeoylquinic acid (4,5-di-CQA), respectively (21-24).

The compounds have been previously isolated from the other species in the genus *Artemisia *such as *Artemisia capillaris*, *Artemisia ciniformis* and *Artemisia annua* (25-27). The presence of these compounds in the genus *Artemisia* has led to valuable effects such as anti-giardial, anti-HBV and inhibition of various enzymes like natural protein tyrosine phosphatase 1B , α-Amylase and α-glucosidase and aldose reductase (28-32). In agreement with the results of the current study, the antioxidant activity of isomers of isochlorogenic acid has been reported in different species of *Artemisia* (33, 34). The results of the Friedman test were consistent with the previous reports for *Artemisia biennis* which suggests none of three antioxidant assays are significantly different in the selection of active extracts/fractions (35). In agreement with the previous reports, a significant correlation was observed between the results of the BCB assay and the total phenolic contents (35, 36). Because of the very low correlation observed between total phenolic content and FIC results, there should have been some other types of phytochemicals which act as secondary antioxidants in the assay. Nitrogen- containing compounds, terpenoids, and polysaccharide fractions as natural non-phenolic antioxidants are some examples which act with different mechanisms (37-41). More studies should be conducted to identify and characterize ferrous ion chelating agents of *A. turanica*.

## Conclusion

This study demonstrated the radical scavenging activity of *A. turanica*, as one of the plant species of Iranian flora. In general, free radical scavenging activities of *A. turanica *hydroethanolic extract and some of its derived fractions in comparison with other samples could be ascribed to their higher content of phenolic compounds like dicaffeoylquinic acids which were isolated in this study. 

## References

[1] Mucciarelli M, Maffei M, Wright CW (2001). Introduction to the genus. Artemisia(Medicinal and aromatic plants- industrial profiles).

[2] Mozaffarian V, Asadi M, Masoumi A (2008). Compositae (Anthemideae & Echinopeae). Flora of Iran.

[3] Mozaffarian V (1996). A dictionary of Iranian plant names. Farhang Moaser Publishers,.

[4] Firouznia A, Akbari MT, Rustaiyan A, Masoudi S, Bigdeli M, Tabatabaei-Anaraki M (2007). Composition of the essential oils of Artemisia turanica Krasch, Helichrysum oocephalum Boiss and Centaurea ispahanica Boiss three Asteraceae herbs growing wild in Iran. J. Essent. Oil Bear. Pl..

[5] Taherkhani M, Rustaiyan A, Taherkhani T (2012). Composition of the leaf essential oils of Artemisiaciniformis Krasch et MvPop ex Poljak, Artemisiaoliveriana J. Gay ex Bess. in DC and Artemisiaturanica Krasch. J. Essent. Oil Bear. Pl..

[6] Ramezani M, Fazli-Bazzaz BS, Saghafi-Khadem F, Dabaghian A (2004). Antimicrobial activity of four Artemisia species of Iran. Fitoterapia.

[7] Emami SA, Vahdati-Mashhadian N, Vosough R, Oghazian MB (2009). The anticancer activity of five species of Artemisia on Hep2 and HepG2 cell lines. Pharmacologyonline.

[8] Emami A, Taghizadeh Rabe SZ, Ahi A, Mahmoudi M (2012). Inhibitory activity of eleven Artemisia species from Iran against Leishmania major parasites. Iran. J. Basic Med. Sci..

[9] Nahrevanian H, Aboufazeli F, Kazemi SM, Hajihosseini R, Naeimi S (2011). Phytochemical evaluation and antimalarial effects of Artemisia turanica herbal extracts as an Iranian flora on plasmodium berghei in-vivo. J. Nat. Remedies.

[10] Taherkhani M, Rustaiyan A, Nahrevanian H, Naeimi S, Taherkhani T (2013). Comparison of antimalarial activity of Artemisia turanica extract with current drugs in-vivo. J. Vector Borne Dis..

[11] Mojarrab M, Naderi R, Heshmati Afshar F (2015). Screening of different extracts from Artemisia species for their potential antimalarial activity. Iran. J. Pharm. Res..

[12] Taherkhani M (2016). Tyrosinase inhibition, in-vitro antimicrobial, antioxidant, cytotoxicity and anticancer activities of the essential oil from the leaves of Artemisiaturanica, growing wild in Iran. J. Essent. Oil Bear. Pl..

[13] Tayarani-Najaran Z, Sareban M, Gholami A, Emami SA, Mojarrab M (2013). Cytotoxic and apoptotic effects of different extracts of Artemisia turanica Krasch on K562 and HL-60 cell lines. Scientific World J..

[14] Hosseinzadeh L, Malekshahi A, Ahmadi F, Emami SA, Hajialyani M, Mojarrab M (2018). The protective effect of different extracts of three Artemisia species against H2O2-induced oxidative stress and apoptosis in PC12 neuronal cells. Pharmacognosy Res..

[15] You Q, Chen F, Wang X, Jiang Y, Yang Z, Sun J, Prasad KN, Ismail A, Yang B, You X (2012). Chemical profiles and health benefits of anthocyanins and phenolic compounds in blueberries. Chemistry, Dietary Sources and Health Benefits (Nutrition and Diet Research Progress).

[16] Lee J, Renita M, Fioritto RJ, St Martin SK, Schwartz SJ, Vodovotz Y (2004). Isoflavone characterization and antioxidant activity of Ohio soybeans. J. Agric. Food Chem..

[17] Singleton VL, Orthofer R, Lamuela-Raventos RM (1999). Analysis of total phenols and other oxidation substrates and antioxidants by means of Folin–Ciocalteau reagent. MethodsEnzymol..

[18] Hatano T, Edamatsu R, Hiramatsu M, Mori A, Fujita Y, Yasuhara T, Yoshida T, Okuda T (1989). Effects of the interaction of tannins with co-existing substances VI: Effects of tannins and related polyphenols on superoxide anion radical, and on 1 diphenyl-2-picrylhydrazyl radical. Chem.Pharm. Bull..

[19] Dinis TC, Maderia VM, Almeida LM (1994). Action of phenolic derivatives (acetaminophen, salicylate, and 5-aminosalicylate) as inhibitors of membrane lipid peroxidation and as peroxyl radical scavengers. Arch. Biochem. Biophys..

[20] Miraliakbari H, Shahidi F (2008). Antioxidant activity of minor components of tree nut oils. Food Chem..

[21] Guo W, Wang L, Gao Y, Zhao B, Wang D, Duan W, Yu Z (2015). Isolation of isochlorogenic acid isomers in flower buds of Lonicera japonica by high-speed counter-current chromatography and preparative high performance liquid chromatography. J. Chromatogr. B Analyt. Technol. Biomed. Life Sci..

[22] Zhu X, Zhang H, Lo R (2004). Phenolic compounds from the leaf extract of artichoke (Cynarascolymus L. ) and their antimicrobial activities. J. Agric. Food Chem..

[23] Timmermann BN, Hoffmann JJ, Jolad SD, Schram KH, Klenck RE, Bates RB (1983). Constituents of Chrysothamnus paniculatus 3: 3, 4, 5-tricaffeoylquinic acid (a new shikimate prearomatic) and 3, 4-, 3, 5-and 4, 5-dicaffeoylquinic acids. J. Nat. Prod..

[24] Puangpraphant S, Berhow MA, Vermillion K, Potts G, Gonzalez de Mejia E (2011). Dicaffeoylquinic acids in Yerba mate (Ilex paraguariensis S Hilaire) inhibit NF-κB nucleus translocation in macrophages and induce apoptosis by activating caspases-8 and-3 in human colon cancer cells. Mol. Nutr. Food Res..

[25] Ko K, Hong IK, Cho H-J, Yang H (2018). Simultaneous determination of four compounds from Artemisiacapillaris using high performance liquid chromatography-ultraviolet detector (HPLC-UVD) and their quantitative study in Artemisia genus. Nat. Prod. Sci..

[26] Nasseri S, Emami SA, Mojarrab M (2019). Dicaffeoylquinic acids from the aerial parts of Artemisiaciniformis Krasch. & Popov ex Poljakov. Pharm. Sci..

[27] Carbonara T, Pascale R, Argentieri MP, Papadia P, Fanizzi FP, Villanova L, Avato P (2012). Phytochemical analysis of a herbal tea from Artemisiaannua L. J. Pharm. Biomed. Anal..

[28] Zhang YH, Xue MQ, Bai YC, Yuan HH, Zhao HL, Lan MB (2012). 3,5-Dicaffeoylquinic acid isolated from Artemisiaargyi and its ester derivatives exert anti-Leucyl-tRNAsynthetase of Giardialamblia (GlLeuRS) and potential anti-giardial effects. Fitoterapia.

[29] Zhao Y, Geng CA, Ma YB, Huang XY, Chen H, Cao TW, He K, Wang H, Zhang XM, Chen JJ (2014). UFLC/MS-IT-TOF guided isolation of anti-HBV active chlorogenic acid analogues from Artemisiacapillaris as a traditional Chinese herb for the treatment of hepatitis. J. Ethnopharmacol..

[30] Zhang J, Sasaki T, Li W, Nagata K, Higai K, Feng F, Wang J, Cheng M, Koike K (2018). Identification of caffeoylquinic acid derivatives as natural protein tyrosine phosphatase 1B inhibitors from Artemisiaprinceps. Bioorg. Med. Chem. Lett..

[31] Olennikov DN, Chirikova NK, Kashchenko NI, Nikolaev VM, Kim SW, Vennos C (2018). Bioactive phenolics of the genus Artemisia (Asteraceae): HPLC-DAD-ESI-TQ-MS/MS profile of the Siberian species and their inhibitory potential against α-amylase and α-glucosidase. Front. Pharmacol..

[32] Jung HA, Islam MN, Kwon YS, Jin SE, Son YK, Park JJ, Sohn HS, Choi JS (2011). Extraction and identification of three major aldose reductase inhibitors from Artemisiamontana. Food Chem. Toxicol..

[33] Zhang L, Tu ZC, Wang H, Wen QH, Fu ZF, Xie X (2016). Antioxidant Activity and Phenolic Acids Profiles of ArtemisiaSelengensis Turcz Extracted with Various Methods by HPLC-QTOF-MS/MS. J. Food Biochem..

[34] Dahmani-Hamzaoui N, Salido S, Linares-Palomino PJ, Baaliouamer A, Altarejos J (2012). On-Line Radical Scavenging Detection and Characterization of Antioxidants from Artemisiaherba-alba. Helv. Chim. Acta.

[35] Hatami T, Emami SA, Miraghaee SS, Mojarrab M (2014). Total phenolic contents and antioxidant activities of different extracts and fractions from the aerial parts of Artemisia biennis Willd. Iran. J. Pharm. Res..

[36] Mojarrab M, Nasseri S, Hosseinzadeh L, Farahani F (2016). Evaluation of antioxidant and cytoprotective activities of Artemisia ciniformis extracts on PC12 cells. Iran. J. Basic Med. Sci..

[37] Drolet G, Dumbroff EB, Legge RL, Thompson JE (1986). Radical scavenging properties of polyamines. Phytochemistry.

[38] Joshi s, Chanotiya CS, Agarwal G, Prakash O, Pant AK, Mathela CS (2008). Terpenoid compositions, and antioxidant and antimicrobial properties of the rhizome essential oils of different Hedychium species. Chem. Biodvers..

[39] Grassmannn J (2005). Terpenoids as plant antioxidants. Vitam. Horm..

[40] Wang J, Zhang Q, Zhang Z, Li Z (2008). Antioxidant activity of sulfated polysaccharide fractions extracted from Laminaria japonica. Int. J.Biol. Macromol..

[41] Chang SC, Hsu BY, Chen BH (2010). Structural characterization of polysaccharides from Zizyphus jujuba and evaluation of antioxidant activity. Int. J. Biol. Macromol..

